# Effect of Magnetic Head Shape on Processing of Titanium Alloy Wire by Magnetic Abrasive Finishing

**DOI:** 10.3390/ma13061401

**Published:** 2020-03-19

**Authors:** Wenlong Li, Yan Chen, Miao Cheng, Yini Lv

**Affiliations:** School of Mechanical Engineering & Automation, University of Science and Technology Liaoning, Anshan 114051, China; lwlhds@163.com (W.L.);

**Keywords:** magnetic abrasive finishing, titanium alloy, abrasive behavior, surface roughness

## Abstract

Titanium alloy wire is characterized by high specific strength, good corrosion resistance, high-temperature resistance and other excellent comprehensive performance. It has been widely used not only in aerospace, shipbuilding and other high-tech fields, but also increasingly in medical equipment, food safety and other fields. Because titanium alloy wire is relatively difficult to process, it has a large deformation resistance, good elasticity, high flexion ratio and more serious rebound. During the processing, adhesion problems may occur, thus reducing the surface quality. The magnetic abrasive finishing (MAF) has good flexible machining characteristics. In this study, the rotating magnetic field was loaded on the titanium alloy wire, and the magnetic abrasive was absorbed by the magnetic field force to form a magnetic abrasive brush, so as to realize the precision processing of the titanium alloy wire. Under the same processing time, when the angle of the magnetic head was 37°, the surface roughness of titanium alloy wire was reduced to 0.28 μm by MAF, which improved the processing quality and efficiency of the titanium alloy wire.

## 1. Introduction

Titanium alloy wire is widely used in medical and health, the military industry and other fields because of its advantages, such as small density, high specific strength and good corrosion resistance. However, due to the limitation of current titanium alloy wire manufacturing, surface defects such as bulges and edges are produced during the reproduction process [1,2,3,4,5]. When titanium alloy wire with defects on the surface is used, it causes instability of the equipment and a certain degree of corrosion in the depression of the titanium alloy wire, thus reducing the service life. Huang et al. [6] obtained better surface integrity by electrolytic polishing of nickel-titanium alloy. However, the electrolyte causes greater pollution to the environment. Krishnan et al. [7,8] used ion implantation to improve the surface quality and to reduce the surface roughness of nickel-titanium wire and titanium wire. However, ion-implantation equipment is highly expensive, the atmosphere used is toxic and the depth of ion implantation is difficult to control. In recent years, some scholars found that magnetic abrasive finishing (MAF) has a significant impact on the surface processing of the difficult-to-machine profiles [9,10]. However, the diameter of titanium alloy wire is too small, making it crucial to ensure that the magnetic abrasive has enough grinding pressure on titanium alloy wire. In the traditional magnetic abrasive finishing process [11,12,13,14,15,16,17,18,19,20,21,22], the square magnetic head is usually used. In the process of polishing titanium alloy wire, due to the small diameter of titanium alloy wire, the pressure of the magnetic abrasive on titanium alloy wire cannot be guaranteed, which affects the processing effect and reduces the processing efficiency. A magnetic head with an angle can change the distribution of the magnetic field in the processing area, and the titanium alloy wire with a smaller diameter can be processed accurately. In addition, a magnetic head with a taper angle can change the distribution of the magnetic induction intensity in the processing area, thus gathering the magnetic abrasive in the area with a high magnetic induction intensity and improving the grinding pressure of the magnetic abrasive on the titanium alloy wire. This eventually helps to realize the precision polishing of the titanium alloy wire [23].

## 2. Principle of Magnetic Abrasive Finishing

### 2.1. Experiment Principle

**Figure 1:**
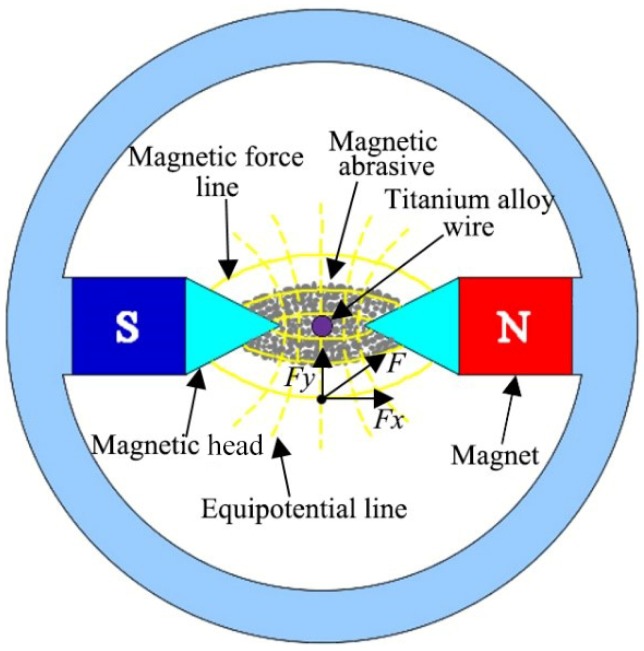
Schematic of processing principle for an magnetic abrasive finishing.

Figure 1 shows the schematic diagram of the titanium alloy wire processed by the magnetic abrasive finishing. Under the action of a magnetic field force, the magnetic abrasive was arranged along the direction of the magnetic field line, and a magnetic particle brush with a certain polishing ability was formed between the two magnetic poles. The resulting magnetic particle brush wrapped the titanium alloy wire to be processed. The magnetic particle brush followed the magnetic pole to make a rotational movement and generated relative motion with the surface of the titanium alloy wire to be processed. The magnetic particle brush continuously cut and scratched the surface of the titanium alloy wire wrapped inside, thereby acting on the titanium alloy wire. The microremoval of the surface material realized the finishing of the surface of the titanium alloy wire and improved the surface quality.

When the titanium alloy wire is being processed, the sum force of the magnetic abrasive in the magnetic field is *F*, which is synthesized by two forces. One is *F_x_* along the tangential direction of the magnetic force line; the other is *F_y_* along the magnetic equipotential line direction.

(1) $$Fx=V\chi \frac{\partial H}{\partial x};Fy=V\chi \frac{\partial H}{\partial y}$$

where *F_x_* is caused by the magnetic field line, along the magnetic field line; *F_y_* is caused by equipotential lines, along the magnetic equipotential lines; *V* is the volume of the magnetic abrasive (m^3^); *χ* is the susceptibility of the magnetic abrasive; and $\frac{\partial H}{\partial x}$ and $\frac{\partial H}{\partial y}$ are the variation rates of the magnetic field strength along the direction of X and Y, respectively.

(2) $$F=\sqrt{F_{x}^{2}+F_{y}^{2}}=V\chi \sqrt{{\left(\frac{\partial H}{\partial x}\right)}^{2}+{\left(\frac{\partial H}{\partial y}\right)}^{2}}$$

where *F* is the total sum force of the magnetic abrasive in the magnetic field; and $\sqrt{{\left(\frac{\partial H}{\partial x}\right)}^{2}+{\left(\frac{\partial H}{\partial y}\right)}^{2}}$ is the magnetic field intensity gradient of the magnetic abrasive finishing in the processing area.

It can be seen from Equation (2) that when the type and amount of magnetic abrasive are given, the magnetic field force of the magnetic abrasive in the magnetic field is positively related to the magnetic field intensity gradient. The impact of the magnetic field intensity gradient on the magnetic abrasive in the magnetic field directly affects the surface quality of the workpiece after machining. Therefore, changing the distribution of the magnetic field intensity in the machining area by adjusting the shape of the magnetic head improves the magnetic field intensity gradient. It is the main method to solve the problem in the magnetic abrasive finishing of titanium alloy wire, which optimizes the surface quality and improves the machining efficiency. In this experiment, we used Maxwell software (16.0, ANSYS, Inc., Canonsburg, PA, USA) to simulate the distribution of magnetic field intensity in the processing area of different shapes of the magnetic head [24]. We then explored the influence of different shapes of the magnetic head on the surface quality and processing efficiency of titanium alloy wire.

### 2.2. Magnetic Field Simulation Analysis

During the processing of titanium alloy wire that has a small size, grinding on thinner wire requires precise control of the effective grinding pressure of the abrasive on the wire. Therefore, it is especially important to ensure sufficient grinding pressure during processing. When the titanium alloy wire is placed in a device that can produce a rotating magnetic field, the magnetic abrasive is arranged along the magnetic field line that passes through the processed titanium alloy wire vertically. Thus, the pressure of the magnetic abrasive on the titanium alloy wire is mainly provided by the magnetic induction intensity gradient. The greater the magnetic induction intensity gradient, the greater the grinding pressure of magnetic abrasive on titanium alloy wire. The magnetic induction intensity gradient is denoted by the change rate of the magnetic field intensity along a certain direction, expressed as:

(3) Gx=ΔB/Δx

where Gx represents the gradient of the magnetic field strength along the x direction; ΔB represents the changes in the magnetic induction strength; and Δx represents the linear distance along the x direction.

Utilization of the magnetic permeability of magnetic heads can guarantee a higher magnetic flux density. A magnetic flux calculation formula is as follows:

(4) φ=BS

where *B* is the magnetic induction intensity; *S* is the area perpendicular to magnetic forcing lines; and *φ* is the magnetic flux through the area *S*.

It can be known from the formula that the magnetic induction intensity is inversely proportional to the area perpendicular to the magnetic force lines. The magnetic head’s area perpendicular to the magnetic force lines can be changed by adjusting the taper of the magnetic head, thereby effectively increasing magnetic induction intensity of the processing area and changing magnetic field distribution. When the angle of the magnetic head is 0°, the magnetic head’s area perpendicular to magnetic force line is the largest; when the angle is 37°, the area is the smallest. The magnetic head of 25°, as one of the choices, was used to serve whether the changes of magnetic induction intensity and magnetic field distribution were the same as the theoretical expectation. Under a premise of constant distance between the magnetic heads, 37° is the max angle that the head can reach. The angle rise will change the distance between the magnetic heads, meaning that a single variable cannot be guaranteed.

**Figure 2:**
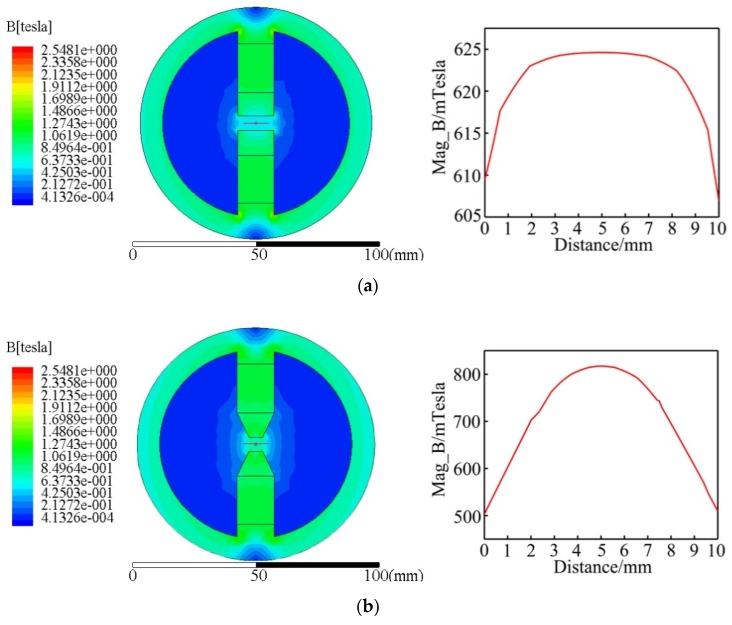

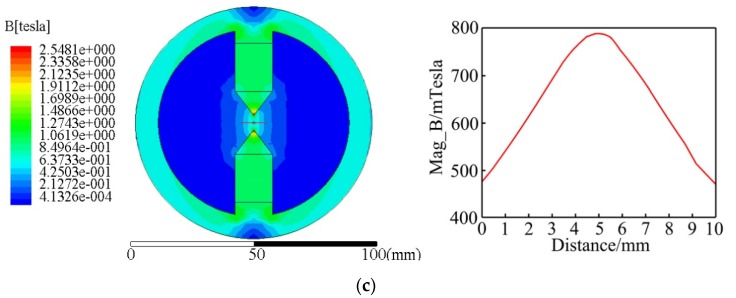
Simulation of the magnetic field intensity and the magnetic induction line: (**a**) 0° taper, distribution diagram; (**b**) 25° taper, distribution diagram; (**c**) 37° taper, distribution diagram.

Maxwell software was used to simulate and analyze the magnetic induction intensity of the processing tools with different shapes of the magnetic head. Figure 2 shows the magnetic field simulation results of different shapes of the magnetic head. Through the analysis and simulation, it was seen that the magnetic induction intensity and magnetic field distribution in the processing area varied with the taper of the magnetic head. The processed wire was perpendicular to the direction of the magnetic-induction line. With the wire as the center, we drew an auxiliary line with a length of 5 mm on both sides to get the change in the magnetic induction intensity of the auxiliary line. As seen from the magnetic induction intensity curve graph of the processing area, when the taper of the magnetic head was 0°, the magnetic induction intensity reached the peak at about 625 mTesla near the wire rod, and tended to be stable, with no obvious change in the magnetic induction intensity gradient. When the taper of the magnetic head was 25°, the maximum magnetic induction intensity near the wire was about 800 mTesla, and the magnetic induction intensity in the processing area varied to some extent. When the taper of the magnetic head was 37°, the maximum magnetic induction intensity was about 800 mTesla, and the magnetic induction intensity varied greatly and the gradient was relatively obvious. From the comparison of the distribution of the magnetic induction intensity of three differently shaped magnetic heads, the theoretical conclusion is drawn as below. When the taper of the magnetic head is 0°, the magnetic induction intensity is weak in the processing area, and the magnetic induction gradient near the titanium alloy wire is low, which cannot provide enough grinding pressure. When the assembled magnetic taper is 25°, the magnetic induction intensity increases at a low rate at a distance of 2 mm away from the wire, and the magnetic induction intensity gradient is small; when far away from the wire, the changes in magnetic induction intensity are more obvious and the magnetic induction intensity gradient increases, but its ability to provide the grinding pressure is still limited. When the taper of the magnetic head is 37°, the magnetic induction intensity is larger and the change of the magnetic induction intensity near the line material is more obvious, and the gradient of the magnetic induction intensity is larger, thus providing a larger grinding pressure.

## 3. Experimental Device and Conditions

### 3.1. Experimental Device

**Figure 3:**
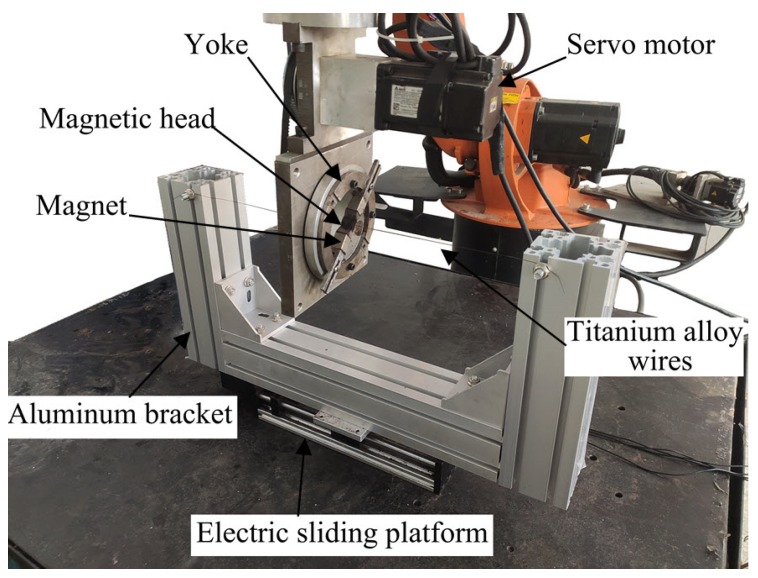
Experimental setup of magnetic abrasive finishing.

Figure 3 is a diagram of the experiment device. The main part of the device that produced the rotating magnetic field used in the experiment was composed of two groups of Nd–Fe–B poles (the pole size is 15 × 15 × 20 mm), two magnetic heads (the material is carbon steel; the size is 15 × 15 × 10 mm), yoke and servo control motor. Two groups of Nd–Fe–B magnetic poles fixed on the yoke produced the required magnetic field in the processing area. The magnetic field intensity and magnetic induction intensity gradient in the processing area can be changed by the magnetic head fixed on the magnetic pole. The processing performance and efficiency can be improved by the adjustment of the magnetic field intensity and the magnetic induction intensity gradient. The servo motor provided the power needed to rotate the magnetic field, and the speed range was 10–1500 rpm. The mobile device was composed of an electric sliding platform, a controller, two fixed supports and titanium alloy wires. The controller controlled the moving speed and range of the electric sliding platform. When being finished, the titanium alloy wire passed through the gap between the two magnetic heads in the pole-rotating device, and was fixed on the electric sliding table through two supports. The processed titanium alloy wire was wrapped between magnets by magnetic abrasive. The electric sliding platform used in the mobile device can make the titanium alloy wire reciprocate between the pole-rotating devices to prevent the forming of an annular groove on the surface of the wire when the same position is processed for a long time.

The titanium alloy wire was passed through the gap between the magnetic head and was fixed on the electric sliding table by a bracket. The electric sliding table drove the titanium alloy wire to move relative to the magnetic particle grinding tool at different speeds. The abrasive used in the experiment was composed of the ferromagnetic phase and the abrasive phase. It is a new type of abrasive with both magnetic and abrasive performance [25,26,27,28]. After the magnetic abrasive was filled between the two magnetic heads, the magnetic abrasive was arranged and combined between the two magnetic poles along the direction of the magnetic line of force to form a magnetic particle brush with the abrasive ability. The abrasive brush formed was tightly wrapped on the surface of the titanium alloy wire to be processed. During the finishing process, the magnetic abrasive tool drove the magnetic abrasive brush to move relative to the surface of the titanium alloy wire to be processed by its own rotation. This helped to realize the microcut of the surface of titanium alloy wire, thus eliminating the irregular edges and bulges on the surface and improving the surface smoothness.

### 3.2. Conditions

The experimental conditions were presented in Table 1. In order to test the influence of magnetic heads with different shapes on the processing of titanium alloy wire by magnetic abrasive finishing, the diameter of titanium alloy wire of 1 mm and the processing length of 300 mm were applied. Magnetic abrasives with magnetic and abrasive properties, which were made by sintering iron particles and Al_2_O_3_ particles with a certain proportion, were employed in this experiment. The abrasive with a particle size of 178 μm and a total weight of 3 g was adsorbed between the magnetic heads by the magnetic force. The rotating speed of the magnetic pole rotating device was 800 rev/min; the titanium alloy wire traveled at 10 mm/s; the distance between the two magnetic heads was 6 mm; and the processing time was 300 s. Due to the small diameter of the workpiece, the oil-based grinding fluid with high viscosity was used as the lapping fluid required by the experiment. The surface microscopic image of the titanium alloy wire was observed by the electron microscope (VHX-500F, Keyence Corporation of America, Itasca, IL, USA), and Alpha-Step IQ (Alpha-Step IQ, KLA-Tencor Corporation, Milpitas, CA, USA) was used to measure the surface roughness with an accuracy of 0.01 μm. The scan length was 500 μm; the scan speed was 50 μm/s; and the sampling rate was 50Hz. In the random measurement of five parts of the samples in the experiment, the average value of the measurement was taken as the final surface roughness value, and the error was reduced by means of the average value of the multipoint measurement.

## 4. Results and Analysis

**Figure 4:**
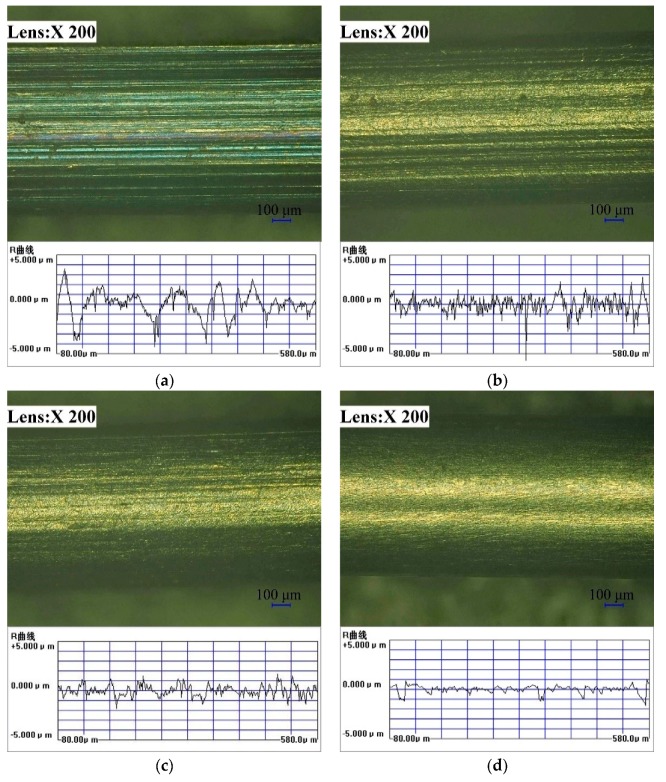
Superfield electron microscopic images of the surface of the titanium alloy wire workpiece before and after magnetic abrasive finishing: (**a**) Before finishing (Ra: 0.92 μm); (**b**) After finishing (Ra: 0.63 μm, magnetic head taper: 0°); (**c**) After finishing (Ra: 0.48 μm, magnetic head taper: 25°); (**d**) After finishing (Ra: 0.28 μm, magnetic head taper: 37°).

Figure 4 shows the microscopic image of the wire. Figure 4a is the original profile of the raw-wire material. From the microscopic image, a layer of black oxide skin on the original surface can be seen. Due to the manufacturing process of the wire itself, there were edges and bulges, and the surface finish was poor. After a period of practical use, there can be a certain degree of wear due to friction, which will affect the stability of the equipment to a certain extent. Figure 4b shows the surface microscopic image after the wire was processed with the 0° polymer head, indicating that the black oxide skin wrapped on the surface of the wire was removed to some extent. Figure 4c shows the surface microscopic image after the wire was processed with the 25° magnetization head, indicating that the black oxide skin wrapped on the surface of the wire was removed completely. Since the edge removal generated by processing and manufacturing was relatively complete, the surface finish was greatly improved. Figure 4d presents the surface microscopic image after the wire was processed with the magnetic head with a taper of 37°, indicating that the black oxide skin on the surface was completely removed.

**Figure 5:**
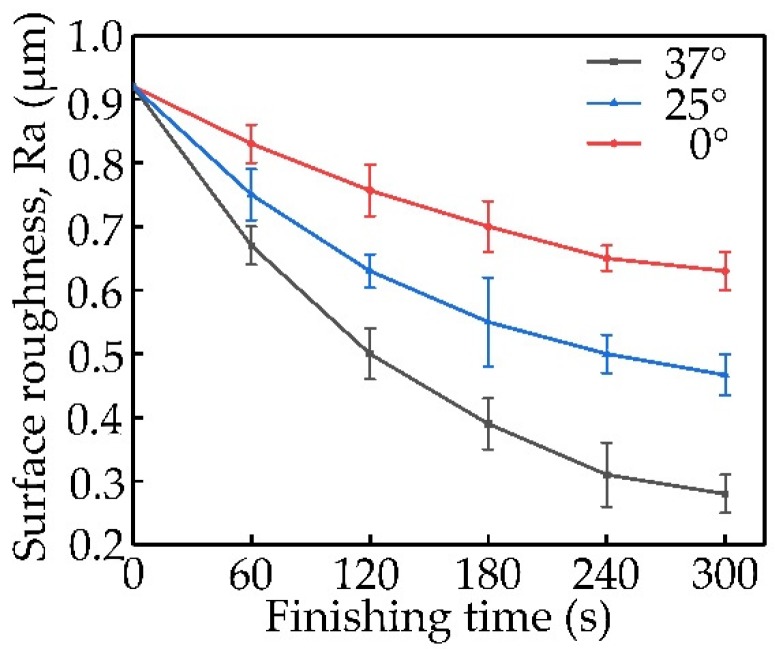
The impact of the shape of the magnetic head on surface roughness.

From Figure 5, the change process of the surface roughness of the wire material processed by the magnetic head with different tapers can be seen clearly. In the early stage of processing, the processing of the magnetic head with different tapers on the wire material was fast, and the change of the surface roughness was large, indicating that the surface roughness dropped fast. Afterward, surface roughness changed slowly, suggesting a low decrease rate of surface roughness. When the wire rod was processed by a magnetic head with a taper of 0°, the change of surface roughness was the lowest in the later stage, and the final value of surface roughness was the lowest among the three. After 300 s of processing, the Ra parameter of the surface roughness reached 0.63 μm. It was seen that when a magnetic head with a taper of 25° was used to process the wire, the change of the surface roughness increased, as well as the change of the surface roughness in the later stage of grinding at a slight rate. After 300 s of processing, the Ra parameter of the surface roughness reached 0.46 μm. When a magnetic head with a taper of 37° was used to process the wire, the surface roughness changed at the highest rate among the three, and finally after 300 s of processing, the Ra parameter of the surface roughness reached 0.28 μm. During the entire grinding-process time, the surface roughness changed quickly. It may be attributed to the difficulty in controlling the grinding pressure on the surface of the wire rod due to the smaller wire diameter. In summary, when the magnetic head with a larger taper was used, there was an obvious change in magnetic induction intensity near the processed wire rod, and the gradient of the magnetic induction intensity was larger. It improved the pressure of the magnetic abrasive on the wire rod surface and promoted the quality and efficiency of magnetic abrasive finishing.

## 5. Conclusions

1. The magnetic induction intensity and magnetic field distribution in the wire-processing area can be changed by the adjustment of the shape of the magnetic head. It affects the processing quality and efficiency of magnetic abrasive finishing on titanium alloy wire.
2. Maxwell software was used to simulate the magnetic induction intensity of differently shaped magnetic heads. The simulation results indicate that when the taper of the magnetic head is 0°, the magnetic induction intensity in the processing area is the lowest, the magnetic induction intensity gradient is low, and the effect after processing is poor; when the taper of the magnetic head is 25°, the magnetic induction intensity in the processing area increases and the magnetic induction intensity gradient changes to some extent, resulting in a better processing effect; when the taper of the magnetic head is 37°, the magnetic induction intensity in the processing area is large and the magnetic induction intensity gradient changes greatly, resulting in the best processing effect.
3. The final of surface roughness was 0.28 μm when the speed of the magnetic pole rotating tool was 800 rev/min, the processing time was 300 s, the abrasive particle size was 178 μm and the taper of the magnetic head was 37°. The grinding quality and efficiency can be improved when the wire rod is processed by the magnetic head with a taper of 37°.
4. Compared with traditional finishing processes, this magnetic abrasive finishing tool has the ability to produce a smooth surface finish, while other polishing processes cannot achieve this goal. Current and future work focuses on designing a method to finish high-precision surfaces of titanium alloy wires. The result demonstrates that this method performs great in polishing surfaces of titanium alloy wires with a diameter of 1 mm. It will also be applied in future work to finish high-precision surfaces of metal wire with a diameter smaller than 1 mm.

## Figures and Tables

**Table 1:** Experimental condition.

Name	Parameter
Workpiece	Titanium alloy wire (Diameter 1 mm, length 300 mm)
Magnetic particle	Sintered (Fe + Al_2_O_3_), 178 μm, 3 g
Grinding fluid	Oil-based grinding fluid
Magnetic head	Q235: 15 × 15 × 10 mm Taper: 37°, 25°, 0°
Magnetic head pitch	6 mm
Magnetic pole rotation	800 rev/min
Titanium alloy wire speed	10 mm/s
Finishing time	300 s
